# Phosphorus addition increases stability and complexity of co-occurrence network of soil microbes in an artificial *Leymus chinensis* grassland

**DOI:** 10.3389/fmicb.2024.1289022

**Published:** 2024-03-27

**Authors:** Xiaoguo Zhou, Yutong Hu, Huijun Li, Jiandong Sheng, Junhui Cheng, Tingting Zhao, Yuanmei Zhang

**Affiliations:** ^1^College of Resources and Environment, Xinjiang Agricultural University, Urumqi, China; ^2^Xinjiang Key Laboratory of Soil and Plant Ecological Processes, Urumqi, China; ^3^The Research Center of Soil and Water Conservation and Ecological Environment, Chinese Academy of Sciences and Ministry of Education, Yangling, China; ^4^University of Chinese Academy of Sciences, Beijing, China; ^5^College of Forestry and Landscape Architecture, Xinjiang Agricultural University, Urumqi, China

**Keywords:** phosphorus addition, *Leymus chinensis*, microbial community, cooccurrence network, keystone species

## Abstract

**Introduction:**

Understanding the response of cross-domain co-occurrence networks of soil microorganisms to phosphorus stability and the resulting impacts is critical in ecosystems, but the underlying mechanism is unclear in artificial grassland ecosystems.

**Methods:**

In this study, the effects of four phosphorus concentrations, P0 (0 kg P ha^−1^), P1 (15.3 kg P ha^−1^), P2 (30.6 kg P ha^−1^), and P3 (45.9 kg P ha^−1^), on the cross-domain co-occurrence network of bacteria and fungi were investigated in an artificial Leymus chinensis grassland in an arid region.

**Results and discussion:**

The results of the present study showed that phosphorus addition significantly altered the stem number, biomass and plant height of the Leymus chinensis but had no significant effect on the soil bacterial or fungal alpha (ACE) diversity or beta diversity. The phosphorus treatments all increased the cross-domain co-occurrence network edge, node, proportion of positively correlated edges, edge density, average degree, proximity to centrality, and robustness and increased the complexity and stability of the bacterial-fungal cross-domain co-occurrence network after 3 years of continuous phosphorus addition. Among them, fungi (Ascomycota, Basidiomycota, Mortierellomycota and Glomeromycota) play important roles as keystone species in the co-occurrence network, and they are significantly associated with soil AN, AK and EC. Finally, the growth of Leymus chinensis was mainly due to the influence of the soil phosphorus content and AN. This study revealed the factors affecting the growth of Leymus chinense in artificial grasslands in arid areas and provided a theoretical basis for the construction of artificial grasslands.

## 1 Introduction

Phosphorus (P) is a crucial mineral element necessary for the growth and development of plants within terrestrial ecosystems (Wang et al., 2017). However, the proportion of phosphorus in the soil that plants can directly absorb and utilize is very low (Heuer et al., 2017; Luo et al., 2019). Many studies have shown that the cooperation of bacteria and fungi can not only promote the absorption of phosphorus by plants but also play a key role in plant phosphorus uptake (Rashid et al., 2016; Anzuay et al., 2017; Ceci et al., 2018). Furthermore, climate change also has an important impact on the healthy growth of plants (Deveau et al., 2018). Microorganisms in the soil demonstrate a remarkable level of sensitivity to changes in their immediate surroundings. These alterations in the environment can directly or indirectly influence the range of species within the microbiome, as well as their diversity in terms of structure and function (Bragazza et al., 2012; Deveau et al., 2018; Guo et al., 2018). It has been shown that under phosphorus addition, there is a significant response from the microbial community (Li et al., 2015 Tian et al., 2015). The exact mechanism through which soil microorganisms interact in artificial grasslands under phosphorus addition conditions has not been determined. Therefore, it is important to explore the response of microbial co-occurrence networks to phosphorus addition and its effect on artificial grasslands to develop a rational fertilization program.

The community of microbial species in the soil environment is not only rich and diverse but also complex (Faust and Raes, 2012). Co-occurrence network analysis is a method for describing complex microbial community structures and can be used to visualize the relationships between microbial communities and reveal symbiotic relationships and influencing factors (Fuhrman, 2009; Zhou et al., 2010). This method has been used to explore microbial interactions in soil environments (Ma et al., 2018). For example, it has been shown that positive interactions in the bacterial-fungal mutualism network are enhanced and negative interactions are reduced after exogenous nutrient addition, which may be due to the fact that nutrient addition enriches the trophic structure of the community and eases competition (Banerjee et al., 2016a). This method can be used to calculate the influential keystone species in the network (Banerjee et al., 2016b). Each node in a co-occurring network can be assigned a role based on its topological attributes, where the topological characteristics are its connectivity within and between network modules. These keystone species are highly connected groups that have significant spatial and temporal effects on the stability, structure, and function of microbial communities (Banerjee et al., 2018; Wagg et al., 2019). These groups not only provide communities with greater biological connectivity but also serve as important indicators of community change. Co-occurrence network complexity can be assessed by correlation network scores such as those derived from connectivity and clustering elements that indicate connectivity among taxa (ASVs). The keystone species in microbial communities are highly important for ecosystems. One study revealed that the keystone species of the microbial community are correlated with soil health and quality (Liu et al., 2022). Notably, long-term fertilization can change the key groups in the co-occurrence network (Lin et al., 2019), and changes in keystone species may lead to changes in the structural and functional diversity of microbial communities (Herren and McMahon, 2018; Fan et al., 2019). For example, keystone species in the microbial community have been shown to be directly related to the rate of soil nitrogen mineralization and to regulate the divergent-convergent trajectory of residue chemistry (Yang et al., 2021; Wang et al., 2023a). Co-occurrence networks are widely used to study microbial community interactions and classify important microorganisms in the soil. However, most related studies have focused on analyzing bacteria and fungi as separate groups, failing to consider the interrelationships between them. Understanding the interactions between bacteria and fungi is crucial for a comprehensive understanding of ecosystem dynamics.

*Leymus chinensis* is a perennial C3 plant that has a strong ability to adapt to saline and drought conditions (Baoyin et al., 2014; Li et al., 2016). Grassland ecosystem protection and restoration have significant potential for managing and enhancing Xinjiang’s grassland ecosystems. In arid regions, we investigated whether phosphorus supplementation affects the composition of soil microbial communities and keystone species, resulting in changes in the structure of the cross-domain bacteria–fungus co-occurrence network and influencing the growth of *Leymus chinensis*. We hypothesized that (1) P addition affects soil environmental factors and the growth of *Leymus chinensis*, (2) P addition changes the co-occurrence network structure, and (3) P addition affects the composition and diversity of keystone species.

## 2 Materials and methods

### 2.1 Study area

Our test site is located at the Sanping Experimental Base of Xinjiang Agricultural University, Urumqi, Xinjiang (87°35′25″E，43°93′31″N, altitude 580 m). Situated in the Eurasian hinterland, the region experiences a prototypical arid continental climate, with an average annual temperature measuring 7.2°C. The temperature can soar as high as 42°C or plummet to as low as −38°C. The area benefits from ample sunlight, with an annual accumulated temperature of approximately 3400°C and a sunshine duration of 2829.4 hours. Precipitation levels average 228.8 mm annually, and evaporation stands at 2647 mm, creating favourable conditions for the cultivation of diverse crops (Li et al., 2023). The tested soil is calcic soil, and the soil organic matter content is 8.12 g·kg^−1^. The content of available phosphorus was 11.22 mg·kg^−1^, available nitrogen was 26.75 mg·kg^−1^, available potassium was 164.25 mg·kg^−1^, and the pH value was 8.2.

### 2.2 Experimental design

*Leymus chinensis* (Zhongke No. 1) seeds were obtained from the Hutubi Experimental Station, which belongs to Xinjiang Jinfangyuan Grassland Ecotourism Development Co., Ltd. and were used as test material in this study. The experimental site was established in October 2019. The phosphorus gradient had four levels: P0 (0 kg P ha^−1^), P1 (15.3 kg P ha^−1^), P2 (30.6 kg P ha^−1^), and P3 (45.9 kg P ha^−1^). The fertilizer used was mono-ammonium phosphate, and the nitrogen treatment was unified at each of the four phosphorus levels (150 kg N ha^−1^); the nitrogen fertilizer used was urea, and each year was divided into spring fertilization and autumn fertilization, of which 50% nitrogen fertilizer was applied in spring and 40% phosphate fertilizer was applied, while the rest were applied in autumn. The plants were randomly distributed, each with an area of 5 m*4 m, and there was a 1 m isolation zone between them.

### 2.3 Sample collection

Soil samples were collected in the third year after *Leymus chinensis* construction (July 12, 2022). A soil auger (with a length of 20 cm and an inner diameter of 4 cm) was used to collect 0–20 cm of soil from each plot, for a total of five points in each plot. Then, the soil was mixed together evenly and partially loaded into 5 mL centrifuge tubes and placed into ice boxes. The samples were subsequently transported to the laboratory and stored at −80°C for DNA extraction. The other part was put into self-sealing bags, labeled for direct return to the laboratory to dry naturally and stored at room temperature for the determination of soil physicochemical properties. A 1 × 1 m square was used to select a representative *Leymus chinensis* plant with uniform growth in the plot, and each experimental treatment was repeated three times. The stem number of *Leymus chinensis* in each square was recorded, the plant height was measured, and then they were killed at 105°C for 30 min and finally dried at 70°C to a constant weight and the weight was recorded.

### 2.4 Index measurement

#### 2.4.1 Soil physical and chemical property indices

At the College of Resources and Environment, Xinjiang Agricultural University, an analysis was conducted on the soil samples to examine their physical and chemical properties. These properties were determined in accordance with the guidelines outlined in the “Soil Agricultural Chemical Analysis” document (Bao, 2000). The values of pH and EC were measured using a potentiometry technique at a soil–water ratio of 1:5. The leaching of sodium bicarbonate and the molybdenum-antimony sulfate resistance colorimetric method were employed to determine the available phosphorus (AP) in the soil. The ammonium acetate extraction method coupled with flame photometry was utilized to determine the available potassium (AK) in the soil. The alkaline hydrolysis diffusion method was employed to determine the available nitrogen (AN), while the molybdenum antimony colorimetric method was used to determine the total phosphorus (TP) concentration. An elemental analyzer (EA3000) was used to determine the total carbon (TC) and total nitrogen (TN) in the soil.

#### 2.4.2 Extraction of soil DNA

We used a TGuide S96 magnetic bead method DNA extraction kit (Tiangen Biochemical Technology (Beijing) Co., Ltd., model: DP812) to complete the extraction of soil nucleic acids according to the manufacturer’s instructions, after which an enzyme labeling instrument (model: synergyHTX) was used to determine the concentration of nucleic acids. DNA samples from bacteria and fungi were analyzed using specific primer sets to amplify the target gene regions. For the bacterial 16S rRNA gene V3-V4 region, the primers 338F (5′-ACTCCTACGGGAGGCAGCA-3′) and 806R (5′-GGACT ACHVGGGTWTCTAAT-3′) were utilized (Quast et al., 2013). In the case of fungi, the region with high variability in the ITS gene was targeted using the primers ITS1F (5′-CTTGGTCATTTAGAG GAAGTAA-3′) and ITS2 (5′-GCTGCGTTCTTCATCGATGC-3′) to amplify the ITS1 region of the 18S rRNA gene (Koljalg et al., 2013). After amplification, the integrity of the PCR products was tested by electrophoresis with agarose at a concentration of 1.8%, and a sequencing library was established quantitatively and uniformly. The established library was first inspected and subsequently sequenced on an Illumina NovaSeq 6000.

#### 2.4.3 Computational analysis

To preprocess the data, we employed Trimmomatic v0.33 software to filter the raw reads obtained from sequencing. Cutadapt 1.9.1 software was subsequently used to detect and eliminate primer sequences, resulting in clean reads devoid of any primer sequences. Then, paired-end sequence splicing was performed with Usearch v10 software to concatenate overlapping clean reads from each sample, and length filtration was applied based on the specific ranges in different regions. Finally, the final valid data (nonchimeric reads) were obtained by denoising and removing chimeric sequences using the dada2 method in QIIME2 2020.6 software (Callahan et al., 2016; Bolyen et al., 2019).

### 2.5 Data analysis

Univariate analysis of variance (LSD for multiple comparisons) was used for soil physicochemical property and microbial alpha diversity data. Beta diversity was analyzed by nonmetric multidimensional scaling (NMDS) with “vegan” in R and Bray–Curtis dissimilarity, and the differences in microbial communities under different phosphorus treatments were tested by ANOSIM. Correlations between keystone species at the phylum level abundance and soil physicochemical properties determined via redundancy analysis (RDA) (performed with Canoco software). Relationships between environmental factors and topological properties of the co-occurrence network were analyzed based on Pearson correlation-based analysis. In addition, we used the “relaimpo” package in R to evaluate the effects of soil physicochemical properties, microbial diversity and keystone species in the bacteria–fungus cross-domain co-occurrence network on the growth of *Leymus chinensis* (Jiao et al., 2019).

The relative abundances of bacteria and fungi were screened before conducting the ecological network analysis. To ensure the accuracy of the network analysis, only the top 0.1% of the bacterial and fungal taxa were chosen based on their relative abundance. Additionally, more than 60% of the soil samples were included in the network analysis (Yang et al., 2022). We analyzed the bacterial-fungal cross-domain co-occurrence network under different fertilization conditions. First, the “phyloseq” package in R was used to construct the data frame for bacterial and fungal ASVs, and the correlation between the ASVs of bacteria and fungi was calculated using the “ggClusterNet” software package (Spearman correlation was used, R2 > 0.9; *p* < 0.05) (Wen et al., 2022). The networks were evaluated by calculating their topological characteristics, including the number of edges, average degree, and average path length, Finally, visualization with Cytoscape 3.9.1. Robustness and natural connectivity are used to determine the stability of the network, and nodes are randomly removed from the constructed co-occurrence network to describe the natural connectivity changes. Robustness was calculated by randomly removing network nodes (50% of nodes were removed) as the ratio between natural connectivity and nonremoval (Deng et al., 2012; Wang et al., 2023). Keystone species were identified as network hubs and module hubs based on the intramodular connectivity (Zi) and intermodular connectivity (Pi) of bacteria-fungi cross-domain co-occurrence networks. Connectors have Pi values higher than 0.62, while module hubs have Zi values greater than 2.5. Conversely, peripherals are characterized by Pi values less than 0.62 and Zi values less than 2.5 (Cong et al., 2015; Shi et al., 2020a).

## 3 Results

### 3.1 Effects of phosphorus on soil physicochemical properties, growth of *Leymus chinensis*

Phosphorus addition significantly increased soil nutrients at 0–20 cm in the artificial *Leymus chinensis* grassland (Table 1). Compared with P0, AP, AN and TP increased with the addition of phosphorus, increasing by 58.02%–137.61%, 2.45–22.47% and 0–24.32%, respectively. Phosphorus addition had no significant effect on the soil pH, EC, or TN (*p* > 0.05). Phosphorus significantly changed the growth of *Leymus chinensis* (Table 2). Compared with P0, phosphorus addition enhanced the number of stems, biomass, and plant height of *Leymus chinensis* in by 43.69%–54.96%, 10.20%–24.04%, and 12.38%–23.04%, respectively.

**Table 1:** Effects of different phosphorus concentrations on soil physical and chemical properties.

Treatment	pH	EC (μS cm^−1^)	AP (mg kg^−1^)	AN (mg kg^−1^)	AK (mg kg^−1^)
P0	8.04 ± 0.06a	223.30 ± 17.63a	15.34 ± 4.08c	46.10 ± 3.63c	171.91 ± 6.149b
P1	8.05 ± 0.02a	188.16 ± 11.98a	24.24 ± 2.93b	47.23 ± 2.49bc	197.63 ± 8.06a
P2	8.08 ± 0.02a	227.23 ± 37.08a	25.02 ± 1.00b	54.15 ± 4.02ab	190.15 ± 23.53ab
P3	8.09 ± 0.01a	199.07 ± 30.67a	36.45 ± 3.26a	56.46 ± 5.67a	177.56 ± 7.40ab

Treatment	TP (g kg^−1^)	TN (g kg^−1^)	TC (g kg^−1^)	C/N
P0	0.37 ± 0.00b	0.14 ± 0.02a	7.33 ± 0.60a	52.64 ± 5.47a
P1	0.37 ± 0.01b	0.14 ± 0.01a	6.89 ± 0.09ab	48.63 ± 2.93ab
P2	0.42 ± 0.01ab	0.15 ± 0.01a	6.50 ± 0.11b	43.75 ± 1.07b
P3	0.46 ± 0.06a	0.15 ± 0.01a	7.60 ± 0.55a	51.84 ± 5.10a

**Table 2:** Effects of different phosphorus concentrations on the stem number, biomass and plant height of *Leymus chinensis*.

Treatment	Stem number (plant/m^2^)	Biomass (g/m^2^)	Plant height (cm)
P0	551.70 ± 11.14b	457.20 ± 15.29b	54.68 ± 1.20c
P1	792.75 ± 33.30a	503.85 ± 9.95ab	61.45 ± 0.73b
P2	810.47 ± 11.89a	559.97 ± 29.62a	62.18 ± 1.31b
P3	854.90 ± 12.08a	567.10 ± 21.09a	67.28 ± 1.61a

### 3.2 Effects of phosphorus treatments on the diversity of soil bacteria and fungi

**Figure 1:**
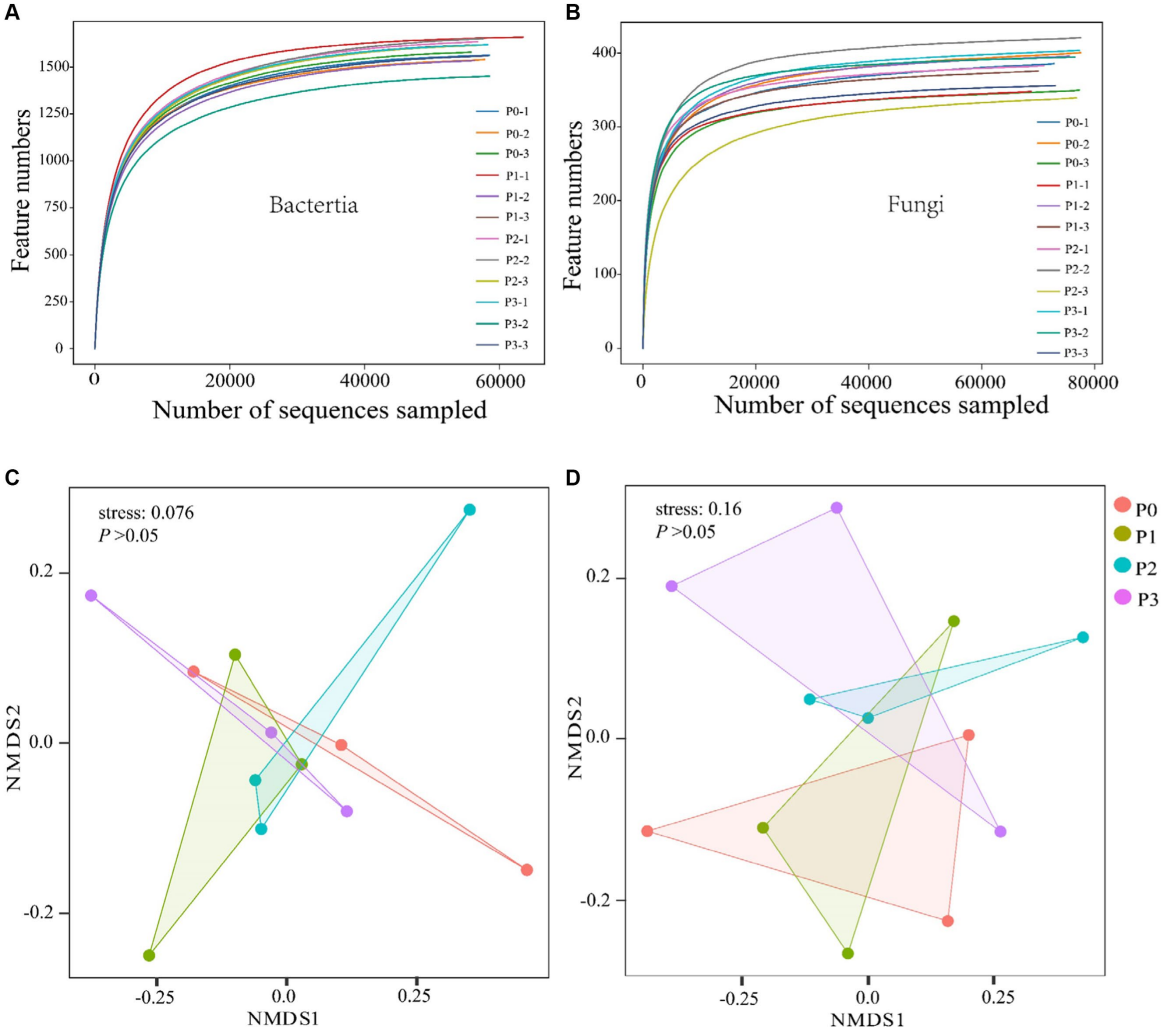
Analysis of soil bacterial and fungal diversity. **(A,B)** Show bacterial and fungal dilution curves. Nonmetric multidimensional scale (NMDS) analysis was conducted using the Bray–Curtis difference method. **(C,D)** Show the differences in bacterial and fungal microbial communities, respectively. ANOSIM was used to test the differences in microbial communities under different fertilization gradients.

Through high-throughput sequencing, 959,619 and 935,153 effective chimeric sequences were obtained for bacteria and fungi from 12 soil samples, respectively, among which 958,122 (99.84%) and 931,844 (99.65%) were high-quality sequences. The OUT dilution curve was used to reflect whether the test results covered all taxonomic groups, and the results of the dilution curve showed that the sequencing data covered all taxonomic groups (Figures 1A,B).

There was no significant effect (*p* > 0.05) on the bacterial or fungal abundance (ACE) or diversity (Simpson) after 3 years of continuous phosphorus addition (Table 3). The overall structure of the soil microbial communities was analyzed based on the Bray–Curtis variability (NMDS) method, which revealed differences in the structure of bacteria and fungi at different phosphorus concentrations; however, according to the ANOSIM test results, phosphorus did not significantly (*p* > 0.05) change the beta diversity of the bacterial and fungal communities (Figures 1C,D).

**Table 3:** Effects of different phosphorus concentrations on the alpha diversity of bacteria and fungi.

	Bacteria		Fungi	
Treatment	ACE	Simpson	ACE	Simpson
P0	1569.41 ± 18.96a	0.998 ± 0.000a	383.35 ± 26.86a	0.956 ± 0.012a
P1	1611.93 ± 60.33a	0.998 ± 0.000a	375.72 ± 23.98a	0.962 ± 0.018a
P2	1646.28 ± 18.83a	0.997 ± 0.001a	384.27 ± 39.91a	0.942 ± 0.043a
P3	1552.56 ± 85.05a	0.997 ± 0.000a	387.01 ± 26.39a	0.963 ± 0.004a

### 3.3 Effect of phosphorus addition on the bacterial-fungal cross-domain co-occurrence network

**Figure 2:**
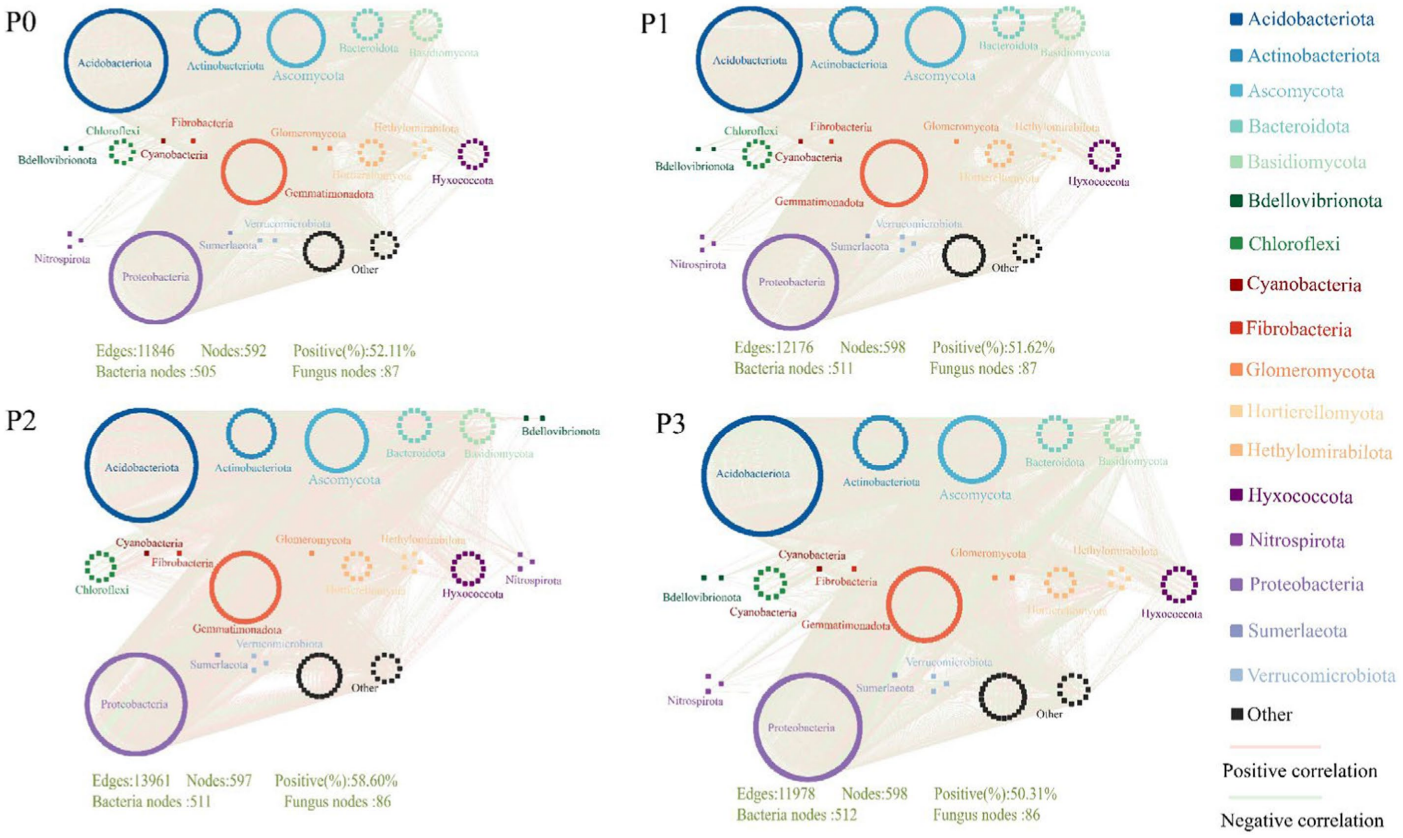
Cross-domain co-occurrence network of bacteria-fungi under different phosphorus level treatments.

**Figure 3:**
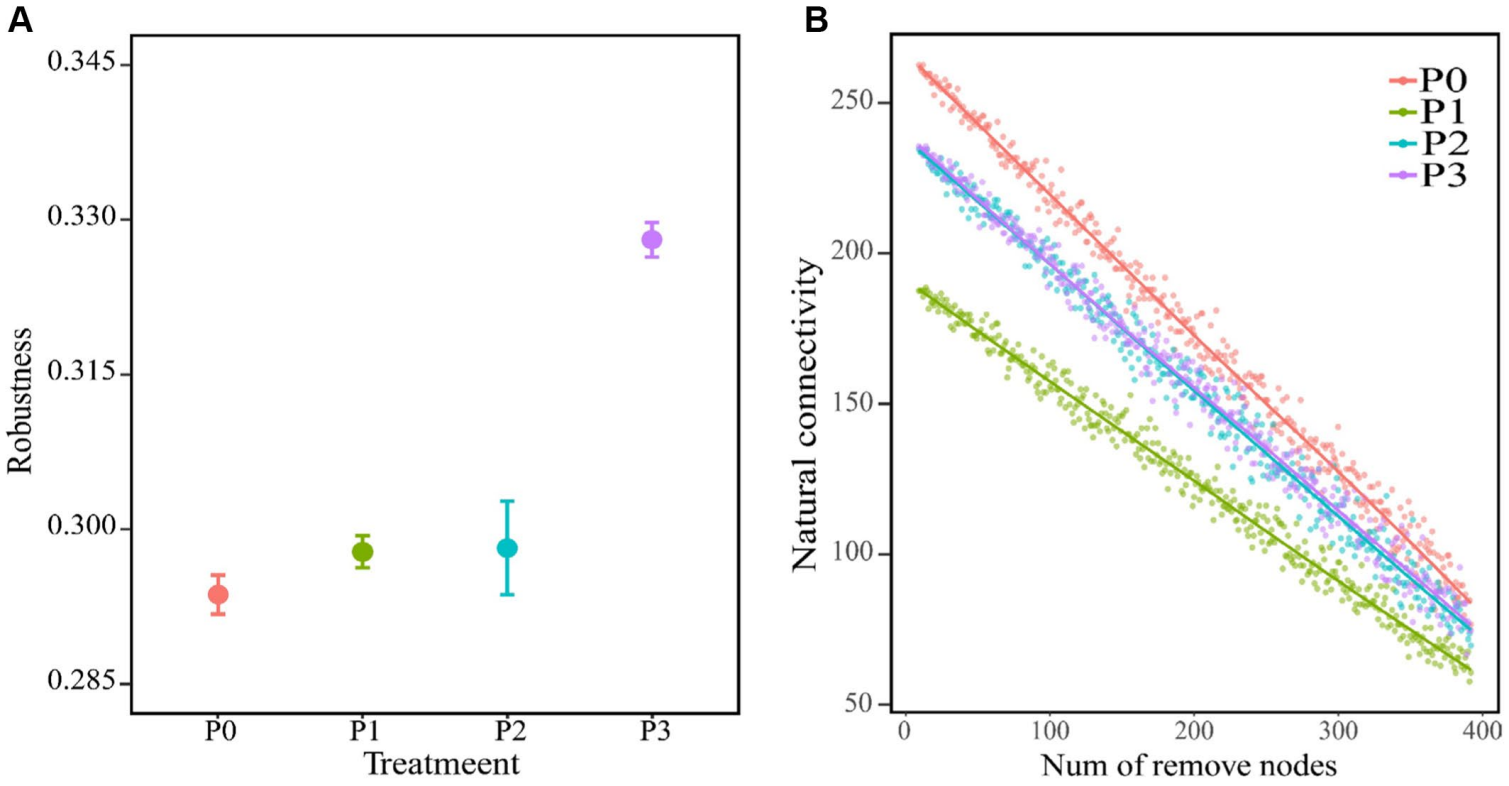
Co-occurring network stability. **(A)** Shows the robustness of the co-occurrence network (where 50% of the nodes are removed), with an average of ±SE and *n* = 3. **(B)** Is the change between the natural connectivity of the co-occurring network and the number of removed nodes.

The complexity of the bacteria–fungi cross-domain co-occurrence network differed significantly under the addition of different levels of phosphorus (Figure 2; Table 4). The P0 co-occurrence network contained 592 edges among 11,846 nodes, and the percentage of positive edges was 52.11%. Edges, nodes and average degree were all greater than those of P0. An elevated topological metric can directly or indirectly indicate an increase in the stability and intricacy of the co-occurrence network or a decrease in its stability and complexity. Stability was also assessed by network robustness natural connectivity, which was highest at P3 (Figure 3A). By calculating the natural connectivity of the network through the randomized removal of nodes, it was observed that the network’s natural connectivity decreased with an increasing number of removed nodes. The highest natural connectivity was achieved at P0 and increased as the concentration of phosphorus added increased (Figure 3B). Among the co-occurrence networks associated with different phosphorus concentrations, we found that the nodes associated with bacteria were much more abundant than those associated with fungi; moreover, the proportion of positively correlated edges first increased and then decreased, and that associated with P2 was the highest.

**Table 4:** Topological characteristics of the bacterial–fungal cross-domain co-occurrence network under phosphorus addition.

Network metrics	P0	P1	P2	P3
Edges	11,846	12,176	13,961	11,978
Num pos edges	6,173	6,285	8,181	6,026
Num neg edges	5,673	5,891	5,780	5,952
Positive (%)	52.11%	51.62%	58.60%	50.31%
Nodes	592	598	597	598
Bacteria nodes	505	511	511	512
Fungus nodes	87	87	86	86
Edge density	0.068	0.068	0.078	0.067
Average degree	40.02	40.72	46.77	40.06
Average path length	1.78	1.75	1.75	1.77
Degree centrality	0.30	0.21	0.26	0.29
Centralization betweenness	0.0053	0.0023	0.0039	0.0035
Centralization closeness	0.65	0.74	0.83	0.64

### 3.4 Relationships between soil physical and chemical properties, keystone species and network topological characteristics

**Figure 4:**
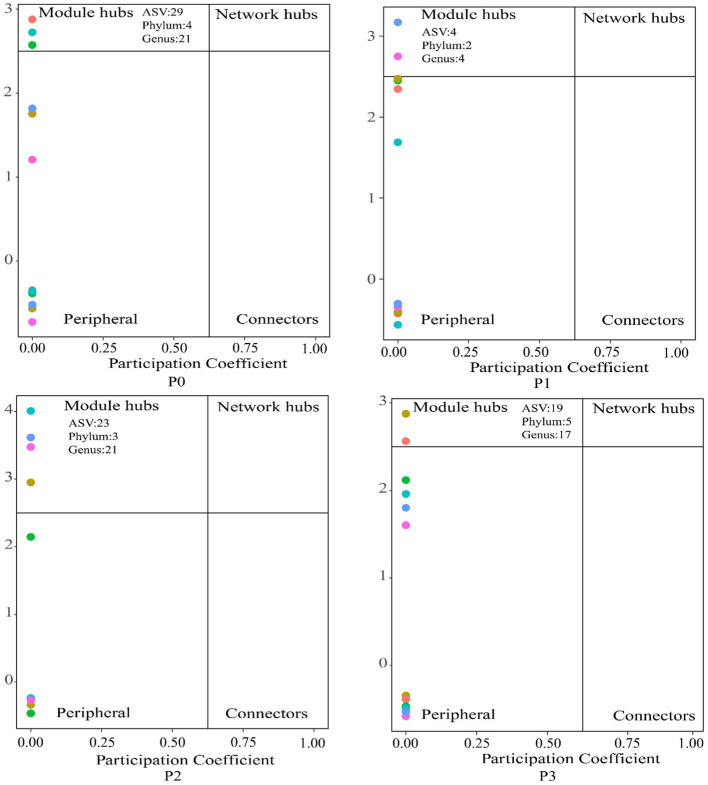
Effects of the addition of different phosphorus levels on the keystone species in the cooccurrence networks. Nodes are classified to identify keystone species in the network based on connectivity within a co-occurring network module (Zi) and connectivity between modules (Pi); connectors s: Pi > 0.62, module network hubs: Pi > 0.62, Zi > 2.5 and module hubs: Zi > 2.5 are identified as keystone species.

**Figure 5:**
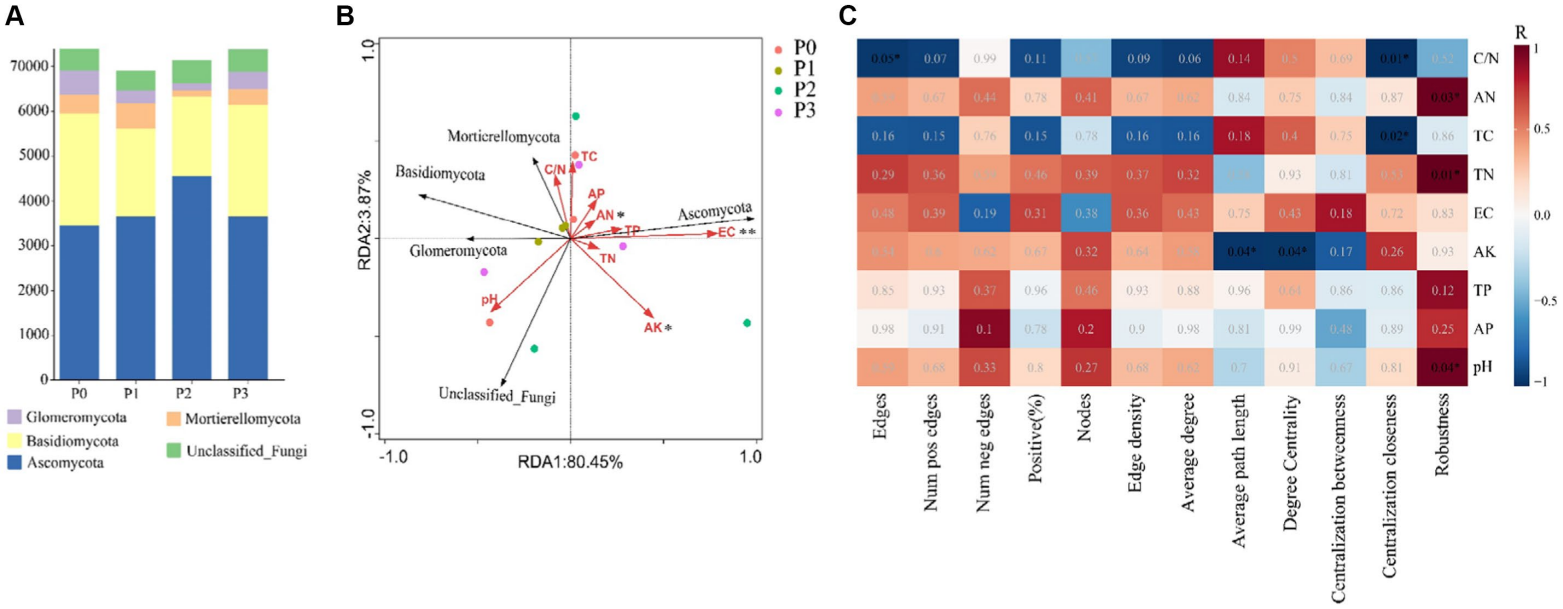
Keystone species abundance, keystone species and environmental factors and network topological characteristics and environmental factor relationships. **(A)** Is the relative richness at the key phylum level. **(B)** RDA of keystone species and environmental factors. **(C)** Based on the relationship between Pearson correlation environmental factors and cooccurrence network topological features. * Indicates that the significance level is *p* < 0.05, ** Indicates that the significance level is *p* < 0.01.

By analyzing the importance of nodes in the dominant network, key nodes (network hubs, module hubs, and connectors) were identified (Figure 4). In this study, we found that keystone species were mainly concentrated in module hubs. These species were found in 56 ASVs in the fungal kingdom, mainly *Ascomycota*, *Basidiomycota*, *Mortierellomycota*, and *Glomeromycotan*. At these taxa levels, we identified 50 keystone genera (Schedule 1). The relative abundance of keystone species increased under phosphorus-addition conditions compared with that under P0 conditions (Figure 5A). The RDA results revealed significant relationships between keystone species and EC (*p* < 0.01) and between AN and AK (*p* < 0.5) (Figure 5B).

Soil physical and chemical properties and co-occurrence network topological characteristics were analyzed based on Pearson correlation, and the results showed that soil C/N pair network edges and centralization closeness, AN, pH, and TN pair robustness, and AK had a significant relationship with average path length and degree centrality, while TC had a significant relationship with centralization closeness (Figure 5C) (Schedule 2).

### 3.5 Effects of soil physical and chemical properties and network keystone species on the growth of *Leymus chinensis*

**Figure 6:**
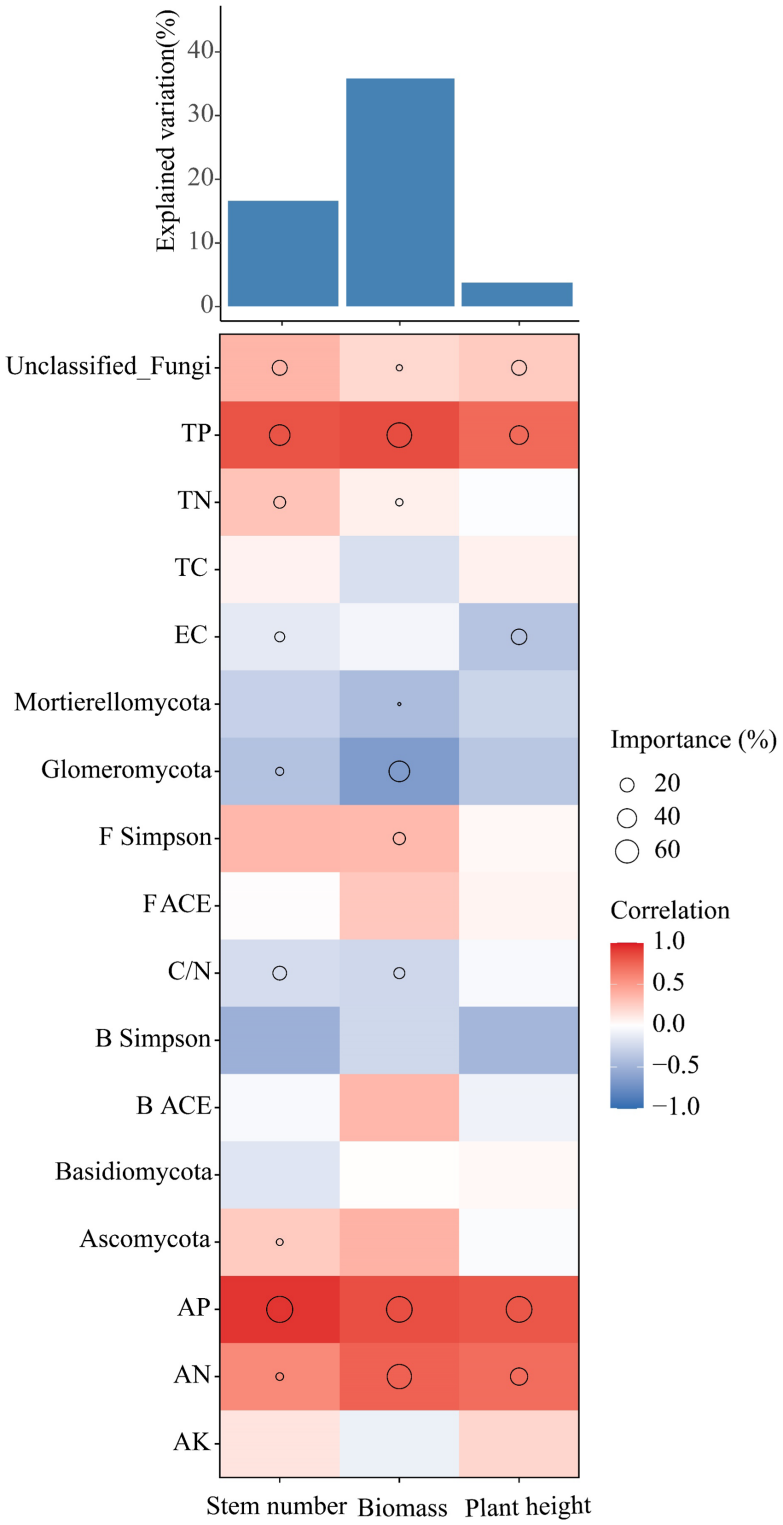
Analysis of soil physicochemical and microbial diversity and the contributions of keystone species in the co-occurrence network to the growth of *Leymus chinensis* based on correlation and optimal multiple regression models. The size of the circle indicates the significance of the variable, and the color represents Spearman’s correlation.

The effects of soil physicochemical properties, microbial diversity and keystone species in the bacterial-fungal cross-domain co-occurrence network on the growth of *Leymus chinensis* were analyzed using multiple regression models (Figure 6). The results showed that soil nutrients were the main factors affecting the growth of *Leymus chinensis* plants under the different phosphorus addition gradients. For example, soil AP, TP and AN were the main variables affecting stem number, biomass and plant height. However, most of the keystone species in the co-occurrence network exhibited a negative correlation with the growth of *Leymus chinensis*. For example, the effects of Glomeromycotan on the stem number and biomass of *Leymus chinensis* were negatively correlated.

## 4 Discussion

### 4.1 Effects of phosphorus on soil physicochemical properties, *Leymus chinensis* growth and microbial diversity

The addition of phosphorus to grassland ecosystems can supplement the nutrients needed by plants, thereby improving soil fertility and productivity. The results of this study showed that pH, AP, AN, TP and TC increased with the addition of phosphorus (Table 1). These results are consistent with those of previous studies (Yu et al., 2009; Zhang et al., 2019; Jiang et al., 2022). These findings may be attributed to the concentration of phosphorus added and residual absorption by *Leymus chinensis* (Zhao et al., 2014; Shi et al., 2020b). In our study, phosphorus significantly affected the growth of *Leymus chinensis* (Table 2) because phosphorus is involved in many metabolic processes during plant growth, such as photosynthesis and other important life processes (Hasan et al., 2016; Li et al., 2016).

In our study, we examined how different levels of phosphorus affect the diversity of soil microbial communities in arid regions. We focused on engineered *Leymus chinensis* ecosystems and analyzed both alpha and beta diversity (Table 2) (Figures 1C,D). Changes in soil phosphorus content can directly or indirectly affect microbial species richness and diversity (DeForest and Scott, 2010; Luo et al., 2015). Our results showed that phosphorus addition did not significantly (*p* > 0.05) affect soil bacterial or fungal abundance (ACE) or diversity (Simpson), consistent with previous results, this is due to the strong buffering capacity of soils, where soil properties stabilize quickly after shorter nutrient additions, so exploring soil microbial changes in response to the environment requires long-term investigations (Xia et al., 2020; Ma et al., 2022). Changes in soil microbial richness may be directly or indirectly caused by changes in environmental factors, such as pH (Gao et al., 2019; Wang et al., 2023b). P has no significant effect on soil pH for three consecutive years according to our results. The different phosphorus treatments had no significant effect on the microbial beta diversity (*p* > 0.05) (Figures 1C,D). It can be inferred that the response of the soil microbial community to phosphorus addition is low (Widdig et al., 2020), this because changes in soil microbial communities under phosphorus conditions are mainly due to nitrate dominance (Xia et al., 2020).

### 4.2 Effects of phosphorus addition on keystone species in co-occurrence networks

Co-occurrence network keystone species can have an enormous impact on other microorganisms and play an important role in maintaining ecosystem function (Berry and Widder, 2014; Ma et al., 2016; Banerjee et al., 2018). Keystone species richness in the co-occurrence network varies under different phosphorus conditions (Figure 5A), the fungal community structure under phosphorus conditions is affected by AN (Huang et al., 2016; Ai et al., 2018; Wu et al., 2023), which is consistent with our results. *Basidiomycota* and *Mortierellomycota* are closely related to soil pH, and phosphorus addition affects soil pH and thus abundance (Deng et al., 2021; Tarin et al., 2021). Some studies have shown that *Ascomycota* can promote soil nutrient cycling (Guo et al., 2020; Chang et al., 2022), this is in line with our findings that its abundance is positively correlated with soil nutrient content (Figure 5B). *Glomeromycotans* belong to a phylum of arbuscular mycorrhizal fungi that promote nutrient uptake (Spatafora et al., 2016; Wipf et al., 2019; Muneer et al., 2020). We speculate that the alteration of soil nutrients directly or indirectly promotes its mutually beneficial relationship with *Leymus chinensis*, thus affecting its richness.

### 4.3 Influence of phosphorus addition on the co-occurrence network

Microbial co-occurrence network analysis can be used not only to determine the interrelationships among groups but also to identify the keystone species that are closely related to the microbial community (Liu et al., 2013; Banerjee et al., 2016a,b). The results of this study showed that phosphorus treatment increased the stability and complexity of the bacteria–fungi co-occurrence networks. We hypothesize that this difference may be attributed to the increase in and accumulation of soil nutrients, which promote cooperation between microbial communities (Johnson, 2010; Trivedi et al., 2020; Lastovetsky et al., 2022). Among the keystone species in this study, *Glomeromycotan* is an arbuscular mycorrhizal fungus (AMF), and interactions between plants and AMF occur through the participation of many microorganisms that have positive effects on plant nutrient uptake. For example, the supply of phosphorus by AMF is facilitated by interactions with phosphorus-soluble bacteria. Phosphorus-soluble bacteria can move along AMF mycelia to reach organophosphorus patches that plants cannot reach (Zhang et al., 2014; Paul et al., 2017; Qian et al., 2019; Jiang et al., 2021). However, in our study, Glomeromycotan abundance was negatively correlated with the growth of *Leymus chinensis* (Figure 6). When Glomeromycotan plants form a symbiotic relationship with the root system of *Leymus chinensis*, they need to rely on the absorption of phosphorus and other nutrients and *Leymus chinensis* nutrient exchange to obtain carbon to maintain normal life activities, and when the phosphorus content in the soil increases, the symbiotic relationship between the two plants will increase (Williams et al., 2016; Qin et al., 2020). The reciprocal and antagonistic relationships between microbial community species can be represented by positive and negative correlation edges, respectively, in the co-occurrence network (Xu et al., 2017). Our study showed that P2 had the highest proportion of positively correlated edges (Table 3), possibly because the addition of phosphorus increased soil nutrients, thereby alleviating competition between microbial communities (Banerjee et al., 2016a,b). In addition, the keystone species in the co-occurrence network under different phosphorus levels were all fungi, which may be due to the long life cycle of fungi and the slow community replacement rate. In addition, changes in soil environmental factors caused by phosphorus addition had a relatively small impact on fungi, and the community had a high diffusion ability (Stegen et al., 2013; Zhou and Ning, 2017).

## 5 Conclusion

The use of microbial co-occurrence networks to determine the response of microbial communities to environmental changes is a novel approach. However, the response mechanism of microbial co-occurrence networks to phosphorus addition in artificial grassland ecosystems and the resulting impact systems are unclear. The results of the present study showed that phosphorus significantly affects the growth of *Leymus chinensis* but has no significant effect on soil bacterial or fungal alpha or beta diversity. The keystone species in the co-occurrence network exhibited significant relationships with AN, AK, and EC, and phosphorus enhanced the stability and complexity of the bacteria–fungi cross-domain co-occurrence network. The growth of artificial *Leymus chinensis* was mainly due to the soil phosphorus content and effects of AN. This study revealed the factors influencing the growth of *Leymus chinensis* in artificial grasslands in arid zones and provided a theoretical basis for the construction of artificial grasslands.

## Data availability statement

The datasets presented in this study can be found in online repositories. The names of the repository/repositories and accession number(s) can be found in the article/supplementary material.

## Author contributions

XZ: Methodology, Investigation, Conceptualization, Data curation, Writing – original draft. YH: Writing – review & editing, Project administration, Funding acquisition, Conceptualization. HL: Writing – original draft, Conceptualization. JS: Supervision, Methodology, Writing – review & editing. JC: Software, Data curation, Writing – review & editing. TZ: Data curation, Writing – original draft. YZ: Writing – original draft, Data curation.

## References

[ref1] Ai C. Zhang S. Zhang X. Guo D. Zhou W. Huang S. (2018). Distinct responses of soil bacterial and fungal communities to changes in fertilization regime and crop rotation. Geoderma 319, 156–166. doi: 10.1016/j.geoderma.2018.01.010

[ref2] Anzuay M. S. Ciancio M. G. R. Luduena L. M. Angelini J. G. Barros G. Pastor N.. (2017). Growth promotion of peanut (*Arachis hypogaea* L.) and maize (*Zea mays* L.) plants by single and mixed cultures of efficient phosphate solubilizing bacteria that are tolerant to abiotic stress and pesticides. Microbiol. Res. 199, 98–109. doi: 10.1016/j.micres.2017.03.006, PMID: 28454714

[ref3] Banerjee S. Baah-Acheamfour M. Carlyle C. N. Bissett A. Richardson A. E. Siddique T.. (2016a). Determinants of bacterial communities in Canadian agroforestry systems. Environ. Microbiol. 18, 1805–1816. doi: 10.1111/1462-2920.12986, PMID: 26184386

[ref4] Banerjee S. Kirkby C. A. Schmutter D. Bissett A. Kirkegaard J. A. Richardson A. E. (2016b). Network analysis reveals functional redundancy and keystone taxa amongst bacterial and fungal communities during organic matter decomposition in an arable soil. Soil Biol. Biochem. 97, 188–198. doi: 10.1016/j.soilbio.2016.03.017

[ref5] Banerjee S. Schlaeppi K. van der Heijden M. G. A. (2018). Keystone taxa as drivers of microbiome structure and functioning. Nat. Rev. Microbiol. 16, 567–576. doi: 10.1038/s41579-018-0024-1, PMID: 29789680

[ref6] Bao S. (2000). Soil agrochemical analysis. 3rd Edn China Agricultural Press.

[ref7] Baoyin T. Li F. Y. Bao Q. Minggagud H. Zhong Y. (2014). Effects of mowing regimes and climate variability on hay production of *Leymus chinensis* (Trin.) Tzvelev grassland in northern China. Rangeland J. 36:593. doi: 10.1071/rj13088

[ref8] Berry D. Widder S. (2014). Deciphering microbial interactions and detecting keystone species with co-occurrence networks. Front. Microbiol. 5:219. doi: 10.3389/fmicb.2014.00219, PMID: 24904535 PMC4033041

[ref9] Bolyen E. Rideout J. R. Dillon M. R. Bokulich N. A. Abnet C. C. Al-Ghalith G. A.. (2019). Reproducible, interactive, scalable and extensible microbiome data science using QIIME 2. Nat. Biotechnol. 37, 852–857. doi: 10.1038/s41587-019-0209-9, PMID: 31341288 PMC7015180

[ref10] Bragazza L. Parisod J. Buttler A. Bardgett R. D. (2012). Biogeochemical plant–soil microbe feedback in response to climate warming in peatlands. Nat. Clim. Chang. 3, 273–277. doi: 10.1038/nclimate1781

[ref11] Callahan B. J. McMurdie P. J. Rosen M. J. Han A. W. Johnson A. J. Holmes S. P. (2016). DADA2: High-resolution sample inference from Illumina amplicon data. Nat. Methods 13, 581–583. doi: 10.1038/nmeth.3869, PMID: 27214047 PMC4927377

[ref12] Ceci A. Pinzari F. Russo F. Maggi O. Persiani A. M. (2018). Saprotrophic soil fungi to improve phosphorus solubilisation and release: *In vitro* abilities of several species. Ambio 47, 30–40. doi: 10.1007/s13280-017-0972-0, PMID: 29159452 PMC5722741

[ref13] Chang Y. Chen F. Zhu Y. You Y. Cheng Y. Ma J. (2022). Influence of revegetation on soil microbial community and its assembly process in the open-pit mining area of the Loess Plateau, China. Front. Microbiol. 13:992816. doi: 10.3389/fmicb.2022.992816, PMID: 36090080 PMC9453671

[ref14] Cong J. Yang Y. Liu X. Lu H. Liu X. Zhou J.. (2015). Analyses of soil microbial community compositions and functional genes reveal potential consequences of natural forest succession. Sci. Rep. 5:10007. doi: 10.1038/srep10007 25943705 PMC4421864

[ref15] DeForest J. L. Scott L. G. (2010). Available organic soil phosphorus has an important influence on microbial community composition. Soil Sci. Soc. Am. J. 74, 2059–2066. doi: 10.2136/sssaj2009.0426

[ref16] Deng Y. Jiang Y. H. Yang Y. He Z. Luo F. Zhou J. (2012). Molecular ecological network analyses. BMC Bioinformatics 13:113. doi: 10.1186/1471-2105-13-113, PMID: 22646978 PMC3428680

[ref17] Deng Q. Zhang T. Xie D. Yang Y. (2021). Rhizosphere microbial communities are significantly affected by optimized phosphorus management in a slope farming system. Front. Microbiol. 12:739844. doi: 10.3389/fmicb.2021.739844, PMID: 34589078 PMC8473901

[ref18] Deveau A. Bonito G. Uehling J. Paoletti M. Becker M. Bindschedler S.. (2018). Bacterial-fungal interactions: ecology, mechanisms and challenges. FEMS Microbiol. Rev. 42, 335–352. doi: 10.1093/femsre/fuy008, PMID: 29471481

[ref19] Fan K. Delgado-Baquerizo M. Guo X. Wang D. Wu Y. Zhu M.. (2019). Suppressed N fixation and diazotrophs after four decades of fertilization. Microbiome 7:143. doi: 10.1186/s40168-019-0757-8 31672173 PMC6824023

[ref20] Faust K. Raes J. (2012). Microbial interactions: from networks to models. Nat. Rev. Microbiol. 10, 538–550. doi: 10.1038/nrmicro2832 22796884

[ref21] Fuhrman J. A. (2009). Microbial community structure and its functional implications. Nature 459, 193–199. doi: 10.1038/nature08058 19444205

[ref22] Gao G. F. Li P. F. Zhong J. X. Shen Z. J. Chen J. Li Y. T.. (2019). *Spartina alterniflora* invasion alters soil bacterial communities and enhances soil N(2)O emissions by stimulating soil denitrification in mangrove wetland. Sci. Total Environ. 653:231. doi: 10.1016/j.scitotenv.2018.10.277, PMID: 30412868

[ref23] Guo X. Feng J. Shi Z. Zhou X. Yuan M. Tao X.. (2018). Climate warming leads to divergent succession of grassland microbial communities. Nat. Clim. Chang. 8, 813–818. doi: 10.1038/s41558-018-0254-2

[ref24] Guo Z. Wan S. Hua K. Yin Y. Chu H. Wang D.. (2020). Fertilization regime has a greater effect on soil microbial community structure than crop rotation and growth stage in an agroecosystem. Appl. Soil Ecol. 149:103510. doi: 10.1016/j.apsoil.2020.103510

[ref25] Hasan M. M. Hasan M. M. Teixeira da Silva J. A. Li X. (2016). Regulation of phosphorus uptake and utilization: transitioning from current knowledge to practical strategies. Cell. Mol. Biol. Lett. 21:7. doi: 10.1186/s11658-016-0008-y, PMID: 28536610 PMC5415736

[ref26] Herren C. M. McMahon K. D. (2018). Keystone taxa predict compositional change in microbial communities. Environ. Microbiol. 20, 2207–2217. doi: 10.1111/1462-2920.14257, PMID: 29708645

[ref27] Heuer S. Gaxiola R. Schilling R. Herrera-Estrella L. Lopez-Arredondo D. Wissuwa M.. (2017). Improving phosphorus use efficiency: a complex trait with emerging opportunities. Plant J. 90, 868–885. doi: 10.1111/tpj.13423, PMID: 27859875

[ref28] Huang J. Hu B. Qi K. Chen W. Pang X. Bao W.. (2016). Effects of phosphorus addition on soil microbial biomass and community composition in a subalpine spruce plantation. Eur. J. Soil Biol. 72, 35–41. doi: 10.1016/j.ejsobi.2015.12.007

[ref29] Jiang Q. Madramootoo C. A. Qi Z. (2022). Soil carbon and nitrous oxide dynamics in corn (*Zea mays* L.) production under different nitrogen, tillage and residue management practices. Field Crop Res. 277:108421. doi: 10.1016/j.fcr.2021.108421

[ref30] Jiang F. Zhang L. Zhou J. George T. S. Feng G. (2021). Arbuscular mycorrhizal fungi enhance mineralisation of organic phosphorus by carrying bacteria along their extraradical hyphae. New Phytol. 230, 304–315. doi: 10.1111/nph.17081, PMID: 33205416

[ref31] Jiao S. Xu Y. Zhang J. Hao X. Lu Y. (2019). Core microbiota in agricultural soils and their potential associations with nutrient cycling. mSystems 4:e00313-18. doi: 10.1128/mSystems.00313-18 30944882 PMC6435817

[ref32] Johnson N. C. (2010). Resource stoichiometry elucidates the structure and function of arbuscular mycorrhizas across scales. New Phytol. 185, 631–647. doi: 10.1111/j.1469-8137.2009.03110.x 19968797

[ref33] Koljalg U. Nilsson R. H. Abarenkov K. Tedersoo L. Taylor A. F. Bahram M.. (2013). Towards a unified paradigm for sequence-based identification of fungi. Mol. Ecol. 22, 5271–5277. doi: 10.1111/mec.12481, PMID: 24112409

[ref34] Lastovetsky O. A. Caruso T. Brennan F. P. Wall D. P. McMahon S. Doyle E. (2022). Evidence of a selective and bi-directional relationship between arbuscular mycorrhizal fungal and bacterial communities co-inhabiting plant roots. Environ. Microbiol. 24, 5378–5391. doi: 10.1111/1462-2920.16227, PMID: 36164274

[ref35] Li J. Cooper J. M. Lin Z. Li Y. Yang X. Zhao B. (2015). Soil microbial community structure and function are significantly affected by long-term organic and mineral fertilization regimes in the North China Plain. Appl. Soil Ecol. 96, 75–87. doi: 10.1016/j.apsoil.2015.07.001

[ref36] Li H. Hu Y. Liu G. Sheng J. Zhang W. Zhao H.. (2023). Responses of biomass accumulation and nutrient utilization along a phosphorus supply gradient in *Leymus chinensis*. Sci. Rep. 13:5660. doi: 10.1038/s41598-023-31402-4, PMID: 37024558 PMC10079846

[ref37] Li L. Yang H. Ren W. Liu B. Cheng D. Wu X.. (2016). Physiological and biochemical characterization of sheepgrass (*Leymus chinensis*) reveals insights into photosynthetic apparatus coping with low-phosphate stress conditions. Journal of Plant Biology 59, 336–346. doi: 10.1007/s12374-016-0117-1

[ref38] Lin Y. Ye G. Kuzyakov Y. Liu D. Fan J. Ding W. (2019). Long-term manure application increases soil organic matter and aggregation, and alters microbial community structure and keystone taxa. Soil Biol. Biochem. 134, 187–196. doi: 10.1016/j.soilbio.2019.03.030

[ref39] Liu X. Liu H. Ren D. Liu C. Zhang Y. Wang S.. (2022). Interlinkages between soil properties and keystone taxa under different tillage practices on the North China Plain. Appl. Soil Ecol. 178:104551. doi: 10.1016/j.apsoil.2022.104551

[ref40] Liu L. Zhang T. Gilliam F. S. Gundersen P. Zhang W. Chen H.. (2013). Interactive effects of nitrogen and phosphorus on soil microbial communities in a tropical forest. PLoS One 8:e61188. doi: 10.1371/journal.pone.0061188, PMID: 23593427 PMC3625167

[ref41] Luo P. Han X. Wang Y. Han M. Shi H. Liu N.. (2015). Influence of long-term fertilization on soil microbial biomass, dehydrogenase activity, and bacterial and fungal community structure in a brown soil of northeast China. Ann. Microbiol. 65, 533–542. doi: 10.1007/s13213-014-0889-9, PMID: 25705148 PMC4331610

[ref42] Luo B. Ma P. Nie Z. Zhang X. He X. Ding X.. (2019). Metabolite profiling and genome-wide association studies reveal response mechanisms of phosphorus deficiency in maize seedling. Plant J. 97, 947–969. doi: 10.1111/tpj.14160, PMID: 30472798 PMC6850195

[ref43] Ma B. Wang H. Dsouza M. Lou J. He Y. Dai Z.. (2016). Geographic patterns of co-occurrence network topological features for soil microbiota at continental scale in eastern China. ISME J. 10, 1891–1901. doi: 10.1038/ismej.2015.261, PMID: 26771927 PMC5029158

[ref44] Ma B. Zhao K. Lv X. Su W. Dai Z. Gilbert J. A.. (2018). Genetic correlation network prediction of forest soil microbial functional organization. ISME J. 12, 2492–2505. doi: 10.1038/s41396-018-0232-8, PMID: 30046166 PMC6155114

[ref45] Ma X. Zhou Z. Chen J. Xu H. Ma S. Dippold M. A.. (2022). Long-term nitrogen and phosphorus fertilization reveals that phosphorus limitation shapes the microbial community composition and functions in tropical montane forest soil. Sci. Total Environ. 854:158709. doi: 10.1016/j.scitotenv.2022.158709, PMID: 36126705

[ref46] Muneer M. A. Wang P. Zaib-un-Nisa Lin C. Ji B. (2020). Potential role of common mycorrhizal networks in improving plant growth and soil physicochemical properties under varying nitrogen levels in a grassland ecosystem. Glob. Ecol. Conserv. 24:e01352. doi: 10.1016/j.gecco.2020.e01352

[ref47] Paul R. Singh R. D. Patra A. K. Biswas D. R. Bhattacharyya R. Arunkumar K. (2017). Phosphorus dynamics and solubilizing microorganisms in acid soils under different land uses of Lesser Himalayas of India. Agrofor. Syst. 92, 449–461. doi: 10.1007/s10457-017-0168-4

[ref48] Qian T. Yang Q. Jun D. C. F. Dong F. Zhou Y. (2019). Transformation of phosphorus in sewage sludge biochar mediated by a phosphate-solubilizing microorganism. Chem. Eng. J. 359, 1573–1580. doi: 10.1016/j.cej.2018.11.015

[ref49] Qin Z. Zhang H. Feng G. Christie P. Zhang J. Li X.. (2020). Soil phosphorus availability modifies the relationship between AM fungal diversity and mycorrhizal benefits to maize in an agricultural soil. Soil Biol. Biochem. 144:107790. doi: 10.1016/j.soilbio.2020.107790

[ref50] Quast C. Pruesse E. Yilmaz P. Gerken J. Schweer T. Yarza P.. (2013). The SILVA ribosomal RNA gene database project: improved data processing and web-based tools. Nucleic Acids Res. 41, D590–D596. doi: 10.1093/nar/gks1219, PMID: 23193283 PMC3531112

[ref51] Rashid M. I. Mujawar L. H. Shahzad T. Almeelbi T. Ismail I. M. Oves M. (2016). Bacteria and fungi can contribute to nutrients bioavailability and aggregate formation in degraded soils. Microbiol. Res. 183, 26–41. doi: 10.1016/j.micres.2015.11.007, PMID: 26805616

[ref52] Shi Y. Delgado-Baquerizo M. Li Y. Yang Y. Zhu Y. G. Penuelas J.. (2020a). Abundance of kinless hubs within soil microbial networks are associated with high functional potential in agricultural ecosystems. Environ. Int. 142:105869. doi: 10.1016/j.envint.2020.105869, PMID: 32593837

[ref53] Shi Y. Ziadi N. Hamel C. Bélanger G. Abdi D. Lajeunesse J.. (2020b). Soil microbial biomass, activity and community structure as affected by mineral phosphorus fertilization in grasslands. Appl. Soil Ecol. 146:103391. doi: 10.1016/j.apsoil.2019.103391

[ref54] Spatafora J. W. Chang Y. Benny G. L. Lazarus K. Smith M. E. Berbee M. L.. (2016). A phylum-level phylogenetic classification of zygomycete fungi based on genome-scale data. Mycologia 108, 1028–1046. doi: 10.3852/16-042, PMID: 27738200 PMC6078412

[ref55] Stegen J. C. Lin X. Fredrickson J. K. Chen X. Kennedy D. W. Murray C. J.. (2013). Quantifying community assembly processes and identifying features that impose them. ISME J. 7, 2069–2079. doi: 10.1038/ismej.2013.93, PMID: 23739053 PMC3806266

[ref56] Tarin M. W. K. Fan L. Xie D. Tayyab M. Rong J. Chen L.. (2021). Response of soil fungal diversity and community composition to varying levels of bamboo biochar in red soils. Microorganisms 9:1385. doi: 10.3390/microorganisms9071385, PMID: 34202337 PMC8306102

[ref3001] Tian W. Wang L. Li Y. Zhuang K. Li G. (2015). Responses of microbial activity, abundance, and community in wheat soil after three years of heavy fertilization with manure-based compost and inorganic nitrogen. Agriculture, Ecosystems & Environment, 213, 219–227. doi: 10.1016/j.agee.2015.08.009

[ref57] Trivedi P. Leach J. E. Tringe S. G. Sa T. Singh B. K. (2020). Plant-microbiome interactions: from community assembly to plant health. Nat. Rev. Microbiol. 18, 607–621. doi: 10.1038/s41579-020-0412-1, PMID: 32788714

[ref59] Wagg C. Schlaeppi K. Banerjee S. Kuramae E. E. van der Heijden M. G. A. (2019). Fungal-bacterial diversity and microbiome complexity predict ecosystem functioning. Nat. Commun. 10:4841. doi: 10.1038/s41467-019-12798-y, PMID: 31649246 PMC6813331

[ref60] Wang Y. Dang N. Feng K. Wang J. Jin X. Yao S.. (2023a). Grass-microbial inter-domain ecological networks associated with alpine grassland productivity. Front. Microbiol. 14:1109128. doi: 10.3389/fmicb.2023.1109128, PMID: 36760496 PMC9905801

[ref61] Wang X. Liang C. Mao J. Jiang Y. Bian Q. Liang Y.. (2023). Microbial keystone taxa drive succession of plant residue chemistry. ISME J. 17, 748–757. doi: 10.1038/s41396-023-01384-2 36841902 PMC10119086

[ref62] Wang D. Lv S. Jiang P. Li Y. (2017). Roles, regulation, and agricultural application of plant phosphate transporters. Front. Plant Sci. 8:817. doi: 10.3389/fpls.2017.00817, PMID: 28572810 PMC5435767

[ref63] Wang Y. Men J. Zheng T. Ma Y. Li W. Cernava T.. (2023b). Impact of pyroxasulfone on sugarcane rhizosphere microbiome and functioning during field degradation. J. Hazard. Mater. 455:131608. doi: 10.1016/j.jhazmat.2023.131608, PMID: 37178534

[ref3002] Wen T. Xie P. Yang S. Niu G. Liu X. (2022). ggClusterNet: An R package for microbiome network analysis and modularity‐based multiple network layouts. iMeta, 1, doi: 10.1002/imt2.32 PMC10989811 38868720

[ref64] Widdig M. Heintz-Buschart A. Schleuss P.-M. Guhr A. Borer E. T. Seabloom E. W.. (2020). Effects of nitrogen and phosphorus addition on microbial community composition and element cycling in a grassland soil. Soil Biol. Biochem. 151:108041. doi: 10.1016/j.soilbio.2020.108041

[ref65] Williams A. Manoharan L. Rosenstock N. P. Olsson P. A. Hedlund K. (2016). Long-term agricultural fertilization alters arbuscular mycorrhizal fungal community composition and barley (*Hordeum vulgare*) mycorrhizal carbon and phosphorus exchange. New Phytol. 213, 874–885. doi: 10.1111/nph.14196, PMID: 27643809

[ref66] Wipf D. Krajinski F. van Tuinen D. Recorbet G. Courty P. E. (2019). Trading on the arbuscular mycorrhiza market: from arbuscules to common mycorrhizal networks. New Phytol. 223, 1127–1142. doi: 10.1111/nph.15775, PMID: 30843207

[ref67] Wu C. Yan B. Wei F. Wang H. Gao L. Ma H.. (2023). Long-term application of nitrogen and phosphorus fertilizers changes the process of community construction by affecting keystone species of crop rhizosphere microorganisms. Sci. Total Environ. 897:165239. doi: 10.1016/j.scitotenv.2023.165239, PMID: 37394065

[ref68] Xia Z. Yang J. Sang C. Wang X. Sun L. Jiang P.. (2020). Phosphorus reduces negative effects of nitrogen addition on soil microbial communities and functions. Microorganisms 8:1828. doi: 10.3390/microorganisms8111828, PMID: 33233486 PMC7699539

[ref69] Xu F. Cai T. Yang X. Sui W. (2017). Soil fungal community variation by large-scale reclamation in Sanjiang plain, China. Ann. Microbiol. 67, 679–689. doi: 10.1007/s13213-017-1296-9

[ref70] Yang F. Chen Q. Zhang Q. Long C. Jia W. Cheng X.. (2021). Keystone species affect the relationship between soil microbial diversity and ecosystem function under land use change in subtropical China. Funct. Ecol. 35, 1159–1170. doi: 10.1111/1365-2435.13769

[ref71] Yang Y. Shi Y. Fang J. Chu H. Adams J. M. (2022). Soil microbial network complexity varies with ph as a continuum, not a threshold, across the North China plain. Front. Microbiol. 13:895687. doi: 10.3389/fmicb.2022.895687, PMID: 35733957 PMC9207804

[ref72] Yu Z.-Y. Zeng D.-H. Jiang F.-Q. Zhao Q. (2009). Responses of biomass to the addition of water, nitrogen and phosphorus in Keerqin sandy grassland, Inner Mongolia, China. J. For. Res. 20, 23–26. doi: 10.1007/s11676-009-0004-4

[ref73] Zhang L. Fan J. Ding X. He X. Zhang F. Feng G. (2014). Hyphosphere interactions between an arbuscular mycorrhizal fungus and a phosphate solubilizing bacterium promote phytate mineralization in soil. Soil Biol. Biochem. 74, 177–183. doi: 10.1016/j.soilbio.2014.03.004

[ref74] Zhang L. Shao H. Wang B. Zhang L. Qin X. (2019). Effects of nitrogen and phosphorus on the production of carbon dioxide and nitrous oxide in salt-affected soils under different vegetation communities. Atmos. Environ. 204, 78–88. doi: 10.1016/j.atmosenv.2019.02.024

[ref75] Zhao S. Qiu S. Cao C. Zheng C. Zhou W. He P. (2014). Responses of soil properties, microbial community and crop yields to various rates of nitrogen fertilization in a wheat–maize cropping system in north-central China. Agric. Ecosyst. Environ. 194, 29–37. doi: 10.1016/j.agee.2014.05.006

[ref76] Zhou J. Z. Ning D. (2017). Stochastic community assembly: does it matter in microbial ecology? Microbiol. Mol. Biol. Rev. 81, e00002–e00017. doi: 10.1128/MMBR 29021219 PMC5706748

[ref77] Zhou J. Deng Y. Luo F. He Z. Tu Q. Zhi X. (2010). Functional molecular ecological networks. MBio 1:1. doi: 10.1128/mBio.00169-10, PMID: 20941329 PMC2953006

