# Supplementary material for: Phosphorus addition increases stability and complexity of co-occurrence network of soil microbes in an artificial Leymus chinensis grassland
Source: Front Microbiol. 2024 Mar 27;15:1289022. doi: 10.3389/fmicb.2024.1289022 (PMC11004269; doi:10.3389/fmicb.2024.1289022)
Supplement: Supplementary file 1 [file Table_1.DOCX]

| OTUID | Phylum | Class | Order | Family | Genus | Species |  |
| --- | --- | --- | --- | --- | --- | --- | --- |
| fun_ASV1 | p__Basidiomycota | c__unclassified_Basidiomycota | o__unclassified_Basidiomycota | f__unclassified_Basidiomycota | g__unclassified_Basidiomycota | s__unclassified_Basidiomycota |  |
| fun_ASV103 | p__Ascomycota | c__Sordariomycetes | o__Hypocreales | f__Nectriaceae | g__Lasionectria | s__Lasionectria_hilhorstii |  |
| fun_ASV11 | p__Ascomycota | c__Sordariomycetes | o__Hypocreales | f__Nectriaceae | g__Fusarium | s__Fusarium_equiseti |  |
| fun_ASV113 | p__Basidiomycota | c__Tremellomycetes | o__Filobasidiales | f__Piskurozymaceae | g__Solicoccozyma | s__Solicoccozyma_aeria |  |
| fun_ASV117 | p__Ascomycota | c__Sordariomycetes | o__unclassified_Sordariomycetes | f__unclassified_Sordariomycetes | g__unclassified_Sordariomycetes | s__unclassified_Sordariomycetes |  |
| fun_ASV124 | p__Ascomycota | c__Pezizomycetes | o__Pezizales | f__Ascodesmidaceae | g__Eleutherascus | s__Eleutherascus_peruvianus |  |
| fun_ASV13 | p__Ascomycota | c__Sordariomycetes | o__Hypocreales | f__Nectriaceae | g__Fusarium | s__unclassified_Fusarium |  |
| fun_ASV131 | p__Ascomycota | c__Dothideomycetes | o__Pleosporales | f__Phaeosphaeriaceae | g__Neosetophoma | s__Neosetophoma_rosigena |  |
| fun_ASV134 | p__Ascomycota | c__Leotiomycetes | o__Helotiales | f__Hyaloscyphaceae | g__Cistella | s__unclassified_Cistella |  |
| fun_ASV135 | p__Ascomycota | c__Sordariomycetes | o__Hypocreales | f__Stachybotryaceae | g__Stachybotrys | s__unclassified_Stachybotrys |  |
| fun_ASV138 | p__Ascomycota | c__Sordariomycetes | o__Hypocreales | f__unclassified_Hypocreales | g__unclassified_Hypocreales | s__unclassified_Hypocreales |  |
| fun_ASV140 | p__Ascomycota | c__Sordariomycetes | o__Hypocreales | Unclassified | g__Acremonium | s__Acremonium_persicinum |  |
| fun_ASV143 | p__Ascomycota | c__Eurotiomycetes | o__Chaetothyriales | f__Trichomeriaceae | g__Knufia | s__unclassified_Knufia |  |
| fun_ASV15 | p__Ascomycota | c__Dothideomycetes | o__Pleosporales | f__Sporormiaceae | g__Preussia | s__Preussia_terricola |  |
| fun_ASV159 | p__Ascomycota | c__Dothideomycetes | Unclassified | Unclassified | g__Bonordeniella | s__Coniosporium_apollinis |  |
| fun_ASV16 | p__Ascomycota | c__Dothideomycetes | o__Pleosporales | f__Phaeosphaeriaceae | g__Neosetophoma | s__unclassified_Neosetophoma |  |
| fun_ASV161 | p__Ascomycota | c__Eurotiomycetes | o__Eurotiales | f__Thermoascaceae | g__Byssochlamys | s__Byssochlamys_spectabilis |  |
| fun_ASV162 | p__Ascomycota | c__Leotiomycetes | o__Helotiales | f__Helotiaceae | g__Tetracladium | s__unclassified_Tetracladium |  |
| fun_ASV18 | p__Ascomycota | c__Sordariomycetes | o__Sordariales | f__Chaetomiaceae | g__Botryotrichum | s__Botryotrichum_domesticum |  |
| fun_ASV21 | p__Basidiomycota | c__Agaricomycetes | o__Agaricales | f__Entolomataceae | g__Entoloma | s__Entoloma_perumbilicatum |  |
| fun_ASV211 | p__Ascomycota | c__Leotiomycetes | o__Thelebolales | f__Pseudeurotiaceae | g__Pseudogymnoascus | s__unclassified_Pseudogymnoascus |  |
| fun_ASV218 | p__Basidiomycota | c__unclassified_Basidiomycota | o__unclassified_Basidiomycota | f__unclassified_Basidiomycota | g__unclassified_Basidiomycota | s__unclassified_Basidiomycota |  |
| fun_ASV22 | p__Ascomycota | c__Dothideomycetes | o__Pleosporales | f__Phaeosphaeriaceae | g__Neosetophoma | s__unclassified_Neosetophoma |  |
| fun_ASV220 | p__Ascomycota | c__Sordariomycetes | o__Microascales | f__Microascaceae | g__Cephalotrichum | s__unclassified_Cephalotrichum |  |
| fun_ASV23 | p__Basidiomycota | c__Agaricomycetes | o__Agaricales | f__Psathyrellaceae | g__Psathyrella | s__unclassified_Psathyrella |  |
| fun_ASV24 | p__Ascomycota | c__Dothideomycetes | o__Cladosporiales | f__Cladosporiaceae | g__Cladosporium | s__unclassified_Cladosporium |  |
| fun_ASV281 | p__Glomeromycota | c__Glomeromycetes | o__Glomerales | f__unclassified_Glomerales | g__unclassified_Glomerales | s__unclassified_Glomerales |  |
| fun_ASV292 | p__Glomeromycota | c__Glomeromycetes | o__Glomerales | f__Glomeraceae | g__unclassified_Glomeraceae | s__unclassified_Glomeraceae |  |
| fun_ASV3 | p__Basidiomycota | c__Agaricomycetes | Unclassified | Unclassified | Unclassified | Unclassified |  |
| fun_ASV318 | p__Basidiomycota | c__unclassified_Basidiomycota | o__unclassified_Basidiomycota | f__unclassified_Basidiomycota | g__unclassified_Basidiomycota | s__unclassified_Basidiomycota |  |
| fun_ASV37 | p__Basidiomycota | c__Agaricomycetes | o__Cantharellales | f__Ceratobasidiaceae | g__Ceratobasidium | Unclassified |  |
| fun_ASV38 | p__Ascomycota | c__Dothideomycetes | o__Pleosporales | f__unclassified_Pleosporales | g__unclassified_Pleosporales | s__unclassified_Pleosporales |  |
| fun_ASV39 | p__Ascomycota | c__unclassified_Ascomycota | o__unclassified_Ascomycota | f__unclassified_Ascomycota | g__unclassified_Ascomycota | s__unclassified_Ascomycota |  |
| fun_ASV4 | p__Ascomycota | c__Dothideomycetes | o__Pleosporales | f__Pleosporaceae | g__Alternaria | s__unclassified_Alternaria |  |
| fun_ASV421 | p__Basidiomycota | c__unclassified_Basidiomycota | o__unclassified_Basidiomycota | f__unclassified_Basidiomycota | g__unclassified_Basidiomycota | s__unclassified_Basidiomycota |  |
| fun_ASV48 | p__Basidiomycota | c__Agaricomycetes | o__Agaricales | f__unclassified_Agaricales | g__unclassified_Agaricales | s__unclassified_Agaricales |  |
| fun_ASV49 | p__Ascomycota | c__Dothideomycetes | o__Pleosporales | f__unclassified_Pleosporales | g__unclassified_Pleosporales | s__unclassified_Pleosporales |  |
| fun_ASV5 | p__Basidiomycota | c__Tremellomycetes | o__Filobasidiales | f__Filobasidiaceae | g__Filobasidium | s__unclassified_Filobasidium |  |
| fun_ASV52 | p__Ascomycota | c__Sordariomycetes | o__Sordariales | f__Chaetomiaceae | g__Chaetomium | s__unclassified_Chaetomium |  |
| fun_ASV54 | p__Mortierellomycota | c__Mortierellomycetes | o__Mortierellales | f__Mortierellaceae | g__Mortierella | s__Mortierella_globalpina |  |
| fun_ASV579 | p__Basidiomycota | c__unclassified_Basidiomycota | o__unclassified_Basidiomycota | f__unclassified_Basidiomycota | g__unclassified_Basidiomycota | s__unclassified_Basidiomycota |  |
| fun_ASV58 | p__Ascomycota | c__Sordariomycetes | o__Sordariales | f__Chaetomiaceae | g__Chaetomium | s__unclassified_Chaetomium |  |
| fun_ASV6 | p__Ascomycota | c__Dothideomycetes | o__Pleosporales | f__Sporormiaceae | g__Sporormiella | s__Sporormiella_megalospora |  |
| fun_ASV61 | p__Ascomycota | c__Eurotiomycetes | o__Chaetothyriales | Unclassified | Unclassified | Unclassified |  |
| fun_ASV7 | p__Ascomycota | c__Sordariomycetes | o__Hypocreales | f__Stachybotryaceae | g__Alfaria | s__unclassified_Alfaria |  |
| fun_ASV79 | p__Ascomycota | c__Sordariomycetes | o__Hypocreales | f__Nectriaceae | g__unclassified_Nectriaceae | s__unclassified_Nectriaceae |  |
| fun_ASV81 | p__Ascomycota | c__Eurotiomycetes | o__Chaetothyriales | f__unclassified_Chaetothyriales | g__unclassified_Chaetothyriales | s__unclassified_Chaetothyriales |  |
| fun_ASV9 | p__Ascomycota | c__Pezizomycetes | o__Pezizales | f__Pyronemataceae | Unclassified | Unclassified |  |
| fun_ASV92 | p__Mortierellomycota | c__Mortierellomycetes | o__Mortierellales | f__Mortierellaceae | g__Mortierella | s__unclassified_Mortierella |  |
| fun_ASV94 | p__Ascomycota | c__Dothideomycetes | o__Pleosporales | f__Cucurbitariaceae | g__Pyrenochaeta | Unclassified |  |
