# Supplementary material for: Phosphorus addition increases stability and complexity of co-occurrence network of soil microbes in an artificial Leymus chinensis grassland
Source: Front Microbiol. 2024 Mar 27;15:1289022. doi: 10.3389/fmicb.2024.1289022 (PMC11004269; doi:10.3389/fmicb.2024.1289022)
Supplement: Supplementary file 2 [file Table_2.DOCX]

| Name | Explains % | Contribution % | F | P |
| --- | --- | --- | --- | --- |
| EC | 50.3 | 56.8 | 10.1 | 0.006 |
| AK | 18.1 | 20.4 | 5.1 | 0.02 |
| AN | 10.2 | 11.6 | 3.8 | 0.028 |
| pH | 4.4 | 4.9 | 1.8 | 0.176 |
| C/N | 0.8 | 1 | 0.3 | 0.834 |
| TP | 0.4 | 0.5 | 0.1 | 0.918 |
| AP | 1.7 | 2 | 0.5 | 0.666 |
| TN | 0.5 | 0.6 | 0.1 | 0.92 |
| TC | 2.1 | 2.3 | 0.4 | 0.698 |
